# Protocol for a comparison study of 1-day (single dose) versus 2-day prophylactic antibiotic administration in Holmium Laser enucleation of the prostate (HoLEP): a randomized controlled trial

**DOI:** 10.12688/f1000research.17660.2

**Published:** 2019-05-09

**Authors:** Katsumi Shigemura, Fukashi Yamamichi, Kento Nishimoto, Koichi Kitagawa, Masato Fujisawa

**Affiliations:** 1 Department of Urology, Kobe University, Kobe, Hyogo, 650-0017, Japan; 2 Department of Infection Control and Prevention, Kobe University Hospital, Kobe, Japan; 3 Department of Public Health, Kobe University Graduate School of Health Science, Kobe, Japan; 4 Department of Urology, Hara Genitourinary Hospital, Kobe, Japan; 5 Division of Advanced Medical Science, Kobe University Graduate School of Science, Technology and Innovation, Kobe, Japan

**Keywords:** HoLEP, Transurethral resection of the prostate, prophylactic antibiotic administration

## Abstract

**Background: **The best method of antimicrobial prophylaxis administration for surgical site infection (SSI) in transurethral holmium laser resection and enucleation of the prostate (HoLEP)/bipolar transurethral enucleation (TUEB) remains controversial. The purpose of this study is to compare one-day and two-day cefazolin in a randomized 2 ^nd^-phase study to help establish a protocol with a 95% confidence interval (CI) for SSI prevention.

**Methods:** Patients undergoing HoLEP/TUEB for benign prostate hyperplasia without preoperative pyuria will be enrolled and randomized to receive prophylactic antibiotic administration for HoLEP/TUEB in two groups, 1-day (single dose) cefazolin and 2-day cefazolin. The primary endpoint is the occurrence rate of postoperative urinary tract infection or urogenital infection within 30 days after HoLEP/TUEB with a statistical 95% CI in comparison between those groups. Secondary outcomes include the kind of infectious disease and evidence of diagnosis, day of diagnosis of infectious disease, performance of urine or blood culture, detection of bacteria, treatments, duration of treatments, AEs other than surgical site infection, and drug-induced AEs.

**Discussion: **The results of this study will provide evidence for defining the optimal duration of cefazolin prophylactic antibiotic administration for SSI.

**Trial registration: **This study was registered in the University Hospital Medical Information Network-Clinical Trial Registry ( UMIN000027955) based on recommendations from the International Committee of Medical Journal Editors (ICMJE) on July 1 ^st^ 2017.

## Abbreviation

SSI: surgical site infection; HoLEP: Holmium laser resection and enucleation of the prostate; TUEB: bipolar transurethral enucleation; CI: confidence interval; TURP: Transurethral resection of the BPH: prostate; benign prostate hyperplasia; UTI: urinary tract infection; PAA: prophylactic antibiotic administration; CEZ: cefazolin

## Trial registration

This study is registered in the University Hospital Medical Information Network-Clinical Trial Registry ( UMIN000027955) based on recommendations from the International Committee of Medical Journal Editors (ICMJE).

## Introduction

Transurethral resection of the prostate (TURP) has been the gold standard for surgical treatment of benign prostate hyperplasia (BPH). However, currently enucleative surgery, such as transurethral holmium laser resection and enucleation of the prostate (HoLEP) or bipolar transurethral enucleation (TUEB), is increasingly performed as a substitution for TURP.

Guidelines for prophylactic antimicrobial administration (PAA) for TURP have been published by the European Association of Urology (EAU), American Urological Association (AUA) and The Japanese Urological Association (JUA) ^1– 3^. Prophylaxis guidelines for HoLEP or TUEB are not fully established ^4^. A meta-analysis by Ahyia *et al.* showed postoperative urinary tract infection (UTI) occurred in 4.1% (0–22%) of TURP cases and 0.9% (0–4.9%) of HoLEP cases, and concluded that HoLEP had a lower rate of SSI ^5^. Similarly, our group presented data from a prospective multi-center study showing that SSI occurred in 8% of TURP cases and 5% of HoLEP cases ^1^. Importantly, no definitive description of the duration of antibiotic dosing was shown and the optimal duration of prophylactic antibiotic administration (PAA) for SSI need to be established.

## Methods

*Study sites*

Patients recruitment began on May 1 ^st^ 2018 and will continue up to April 30 ^th^ 2021 from the institutions of Kobe University Hospital, Kobe University International Clinical Cancer Research Center, Hara Genitourinary Hospital, Shinko Memorial Hospital, Hyogo prefectural Amagasaki General Medical Center, Kobe City Medical Center West Hospital, Kakogawa Central City Hospital and Hyogo Prefectural Kakogawa Medical Center. Those patients undergoing HoLEP/TUEB for BPH without preoperative pyuria will be enrolled. Preoperative pyuria will be defined as 5 or more white blood cells (WBC)/higher power field (HPF). Since PAA duration is limited to 72 h or less in TURP and 48h or less in HoLEP or TUEB ^6^, we will carry out a randomized study of 1-day (single dose) and 2-day PAA for HoLEP/TUEB using cefazolin (CEZ). This is a feasible randomized 2 ^nd^ phase study to help design further confirmatory studies evaluating the differences of SSI occurrence rate with a 95% confidential interval.

### Eligibility criteria

*Selection criteria*

In the period from May 1 ^st^ 2018 to April 30 ^th^ 2021, patients 20 years old or older undergoing HoLEP/TUEB without preoperative bacteriuria will be enrolled.

*Exclusion criteria*

i) Patients who have undergone another procedure such as prostate biopsy or bladder urolithiasis at the time of HoLEP/TUEB. ii) Those with an indwelling urethral catheter. iii) Those with an allergy to CEZ. iv) Hemodialysis patients.

### Interventions

Eligible patients will be randomized in equal proportions between 1-day (single dose) and 2-day PAA for HoLEP/TUEB using cefazolin (CEZ).

### Outcomes

*Primary endpoint*

Primary endpoint is to compare SSI occurrence rate in both 2 groups.

Items of postoperative infection complications:

30-days postoperative infectious complications after HoLEP

In this study, UTI and urogenital infection means prostatitis, epididymitis, pyelonephritis and urosepsis. The occurrence date of these infections and the duration (days) until disappearance of pyuria, in those cases with pyuria, will be recorded.

*Secondary endpoints*

In cases where perioperative infection requires antibiotic therapies, the following information will be recorded. 1) The kind of infection and reasons for the diagnosis; 2) Occurrence data of such infection; 3) Blood culture and urine culture; 4) Identified bacteria; 5) Methods of therapies; 6) Duration of therapies; 7) Other postoperative complications than infectious ones; 8) Drug-induced adverse events.

*Feasible purpose*

This is a feasible randomized study to investigate the occurrence rate of postoperative UTI or urogenital infection within 30 days after HoLEP/TUEB with an estimated 95% CI, and will be followed by confirmatory studies.

## Participant timeline (See Table 1)

**Table 1.** Participant timeline.

Items		Preoperative observation period	Day of dosing (surgery day)	Postoperative observation period				
Term		Hospitalized day	0 week	1 ^st^ day after administration	2nd day after administration	1 week after administration	2 weeks after administration	4 weeks after administration
Consultation		During hospitalization	During hospitalization	During hospitalization	During hospitalization	During hospitalization	Consultation2	Consultation3
Informed consent		○						
Confirmation of patients’ background		○						
Antibiotic administration (one-day group)			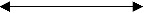					
Antibiotic administration (2-days group)			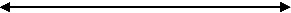					
Subjective symptom/objective findings		○	○	●	●	●	●	●
Observation of adverse events including infectious complications ^a^			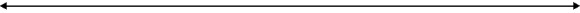					
Blood pressure		○	○	●	●	●		
Heart rate		○	○	●	●	●		
Body temperature		○	○	●	●	●	●	●
Laboraty tests	Hematological examination	○	○	●		●	●	●
	Biochemical examination	○	○	●		●	●	●
	Urinalysis ^d^	○	○	●		●	●	●
Chest X-ray		○						
ECG		○						
Urine culture		○	○					

○; Items to be done before antibiotic administration, •: Items to be done after antibiotic administration

a: Adverse events are not necessarily associated with antibiotics.

b: Hematological examination consists of hematological laboratory tests, biochemical laboratory tests and urinalysis to check the safety of the tested antibiotics and includes white blood cell (WBC) and differential white blood count as inflammatory markers. The volume for hematological laboratory tests is 8ml.

c: Biochemical laboratory tests use CRP as inflammatory markers, which is performed within the range of daily clinical examination.

d: Urinalysis includes white blood cell (WBC) and bacteriuria as inflammatory markers. These tests are done as a confirmation safety check for this study.

### Sample size

For a feasible randomized comparing study, the target sample size is n=180 (1 day (single dose): n=90 and 2 days: n=90). The sample calculation was performed as follows: this study is a feasibility study. We referred to the following study which examined their 164 TURP and HoLEP cases, and found the postoperative infectious complications in 7/72 cases (9.7%) and 2/72 cases (2.8%), respectively. Accordingly, if we estimate 3 or 4 cases of UTI or urogenital infectious complication within 30 days after HoLEP, the occurrence frequencies for a 95% confidence interval (CI) are 0.007-0.094 and 0.012-0.111, respectively. The upper limit of a 95% CI is 10% or so, and it may be useful for planning the next study to set them as in Jhanwar *et al.* ^7^.

*Statistical analysis*

Analysis of study participants’ background:

The difference between groups will be analyzed by the following methods:

Pearson’s chi-square: nominal variables; Fisher’s direct probability calculation method is performed where the expected frequency of less than 5 is 20% or higher; T-tests will be done for continuous variables. The Significance standard is set as 5% in two-sided tests.

### Recruitment strategy

Recruitment will be performed from the patients with indication of HoLEP in those institutions participated in this study. Randomization will be performed by a table of random numbers as a simple randomized study under the control of the responsible party (Dr. Yuzo Nakano, Department of Urology, Kobe University Hospital).

### Allocation

Participants will be randomly assigned to either 1-day or 2-day antibiotic group with a 1:1 allocation as per a computer-generated randomization schedule.

### Blinding

Assessments regarding clinical recovery will be conducted by Dr. Yuzo Nakano blind to treatment allocation. Due to the nature of the intervention neither participants nor staff can be blinded to allocation, but are strongly inculcated not to disclose the allocation status of the participant at the follow up assessments. An employee outside the research team will feed data into the computer in separate datasheets so that the researchers can analyse data without having access to information about the allocation

### Assignment of intervention

*Tested antibiotics*

1 ^st^ generation cephalosporine : Cefazolin Sodium Hydrate (J01DB04)

*Method of administration*

Patients will be randomly divided into a 1-day (single dose) group (CEZ 1g, once per i.v. just before HoLEP/TUEB) and a 2-day group (CEZ 1g, i.v. just before HoLEP/TUEB with a repeat dose the next morning). Comparison of SSI occurrence in these 2 groups is a feasible randomization study.

*Outline of study*

i) One-day dosing (single dose): i.v. initiation of CEZ (1g) 30 min prior to surgery, completed in 30–60 min (with no any other antibiotic administration)

ii) Two-day dosing: i.v. initiation of CEZ (1g) 30 min prior to surgery, completed in 30–60 min, repeated every 12 hours for 2 days including the surgery day. Additional dosing is necessary in cases with 3 hours or longer of surgery time. Test items and schedule of this study is shown in Table 1.

*Study therapy*

To investigate the inhibiting effect of cefazolin (CEZ) 1-day (single dose) and 2-day administration on perioperative infectious complications in HoLEP with a calculated 95% CI.

*Combination medicine*

Exclusion criteria include use of other antibiotics or cases requiring an additional antibiotic. 

*Termination of antibiotic administration*

Cases exhibiting or suspicious for drug-induced allergy.

### Assignment of interventions (for controlled trials)

*Management and delivery of study drug*

Applicants will be contacted by fax or e-mail and then given either 1-day (single dose) or 2-day CEZ under randomization as described above.

### Post-test treatments

In cases where the attending physician diagnoses a perioperative infectious complication, the doctor can treat at discretion, including i.v. for severe cases and oral antibiotics for mild cases.

### Evaluating items

We will gather the following data from the medical records.

i) Patients’ background factors

Age, Body Mass Index (BMI), Preoperative IPSS/QOL・Qmax・residual urine volume (ml), Estimated prostate volume(ml), Preoperative PSA, ASA-PS, Diabetes mellitus (HbA1c, blood sugar control), Chemotherapy and immune-suppressants

ii) Surgery-related categories

Surgical time (min, including morcellation), Resected prostate weight (g), Catheterized period (days), Post-operative residual urine volume (ml): until 30 days after surgery, Duration of antibiotic administration (1 day (single dose) or 2 days)

iii) Postoperative infectious complications

Investigation for 30 days after surgery (need to record), UTI or urogenital infection (prostatitis, epididymitis or pyelonephritis), Postoperative complication other than infectious ones, Occurrence date, Cases with infectious complications requiring additional antibiotic therapy, Detail of infection and the diagnosing evidence, Infection occurrence date, Urine culture and blood culture, Detected bacteria, Therapy, Therapeutic duration, and Days needed to reduce pyuria

### Screening tests

Screening tests will be done to check the following criteria i) and ii).

i) Enrollment criteria

Those patients who are older than 20 years old undergoing HoLEP for benign prostate hyperplasia (BPH) without bacteriuria preoperatively and with informed consent.

ii) Exclusion criteria: see above

### Information on participants

The following information will be recorded at the time of informed consent acquisition and screening tests.

i) Date of informed consent acquisition; ii) Class card number of study participants; iii) Backgrounds of study participants, birth date, age, height, body weight, past history, complication (underlying disease), iv) Present illness: date of definitive diagnosis, risk scoring, family history

## Items for observation/ tests/ evaluation

### Subjective symptom/ objective findings

Observation of adverse events including infectious complication, blood pressure, heart rate, body weight, hematology examination, blood biochemical examination, urinalysis, chest X-ray, ECG, urine culture

### Patients’ background

Age, body mass index (BMI), preoperative PSS/QOL・Qmax・residual urine volume (ml) estimated prostate weight (ml), preoperative PSA, ASA-PS, diabetes mellitus (HbA _1c_, blood sugar control), chemotherapy or immune-suppressants,

### Surgery-related items

Surgical time (min; including morcellation time), resected prostate weight (g), duration (days) to removal of urethral catheter postoperatively, Postoperative residual urine volume (ml) till 30-days after surgery, duration of prophylactic antibiotic administration (CEZ) : 1-day (single dose) or 2-days group

### Items for postoperative perioperative infection and complications: Examination till 30-days after surgery

UTI and urogenital infection (prostatitis, epididymitis and pyelonephritis), postoperative adverse events including infectious ones, except postoperative infection, occurrence date.

In cases with perioperative infection who need antibiotic therapies, the details include bacterial information (see above).

### Cancelation criteria

i) Those cases who are not willing to continue this study and/or wish to withdraw informed consent. ii) Those cases who are found not to be satisfied about their applicability to this study or who cannot continue the study owing to the occurrence of complications and/or exacerbation. iii) Those cases who cannot continue the study owing to study-related adverse events. iv) The study itself is canceled. v) Cases with uncontrollable infectious complications postoperatively. vi) PIs or the members of this research judge that cancelation is needed owing to other reasons.

### Study period

May 1 ^st^ 2018 to April 30 ^th^ 2021; the follow-up period is 1 year afterwards.

## Analysis of efficacy

### Main analysis

We will estimate the difference in postoperative infection occurrence rate at the 95% CI and do the 5% two-sided test for Significant for such differences.

### Interim analysis

This study needs interim analysis because it is planned as a feasible randomized study.

That is, once 45 cases are completed, the occurrence rate of postoperative infection can be compared in the 2 groups and to determine if there is less than a 5% of difference and then to continue if the difference is 5% or less. 

### Ethics and dissemination

*Ethical consideration and consent*

This protocol was approved by the Institutional review board of the Kobe University Graduate School of Medicine (C180043). All procedures were in compliance with the relevant laws and guidelines in accordance with the ethical standards of the Declaration of Helsinki.

This study was registered in the University Hospital Medical Information Network-Clinical Trial Registry ( UMIN000027955) based on recommendations from the International Committee of Medical Journal Editors (ICMJE) on July 1 ^st^ 2017.

***Protocol amendments***

Any modifications to the protocol which may impact on the conduct of the study, potential benefit of the patient or may affect patient safety, including changes of study objectives, study design, patient population, sample sizes, study procedures, or significant administrative aspects will require a formal amendment to the protocol. Such amendment will be approved by the Ethics Committee/IRB (institutional review board) prior to implementation and notified to the health authorities in accordance with local regulations. Administrative changes of the protocol are minor corrections and/or clarifications that have no effect on the way the study is to be conducted. These administrative changes will be agreed upon by Ethics Committee/IRB, and will be documented in a memorandum. The Ethics Committee/IRB may be notified of administrative changes at the discretion of Helsinki Declaration.

### Consent of assent

A trained research doctor will introduce the trial to patients who will be shown an informed consent form regarding the main aspects of the trial. Research doctors will discuss the trial with patients in light of the information provided in information sheets. Patients will then be able to have an informed discussion with the participating consultant. Research doctors will obtain written consent from patients willing to participate in the trial. Information sheets and consent forms are provided for all patients (Extended data ^8^). All information sheets and consent forms transcripts have been translated into Japanese.

### Confidentiality

All study-related information will be stored securely at the study site. All participant information will be stored in locked file cabinets in areas with limited access. All laboratory specimens, reports, data collection, process, and administrative forms will be identified by a coded ID number only to maintain participant confidentiality. All records that contain names or other personal identifiers, such as locator forms and informed consent forms, will be stored separately from study records identified by code number. All local databases will be secured with password-protected access systems. Forms, lists, logbooks, appointment books, and any other listings that link participant ID numbers to other identifying information will be stored in a separate, locked file in an area with limited access.

### Access to data

The Data Management Coordinating Center will oversee the intra-study data sharing process, with input from the Data Management Subcommittee.

All Principal Investigators will be given access to the cleaned data sets. Project data sets will be housed on the Project Accept Web site and/or the file transfer protocol site created for the study, and all data sets will be password protected. Project Principal Investigators will have direct access to their own site’s data sets, and will have access to other sites data by request. To ensure confidentiality, data dispersed to project team members will be blinded of any identifying participant information.

### Ancillary and post-trial care

Patients that are enrolled into the study are covered by national health insurance in all the tests and treatments including the ones performed additionally owing to the adverse events of this study.

### Dissemination policy

We plan to disseminate the information of this study through the UMIN and our department homepage.

### Study status

The study is now undergoing pre-recruitment for participants (recruitment started May 1 ^st^, 2018).

## Discussion

This study is designed to address the following issues:

1) AMR: antimicrobial resistance action plan

WHO suggested the AMR action plan in 2015 based on recent trends of emergence of antibiotic resistant bacteria in infectious diseases. Many researchers suggest that inappropriate antibiotics use may cause this problem. Inappropriate antibiotic prescriptions are often seen, for instance in upper respiratory infections which is caused by not only bacteria but also virus ^9^. Accordingly, the Japanese government suggested an AMR action plan aiming at a 50% decrease in the use of oral antibiotic such as cephalosporines or macrolides ^10^. The chief justification for the preventive use of antibiotics is for the purpose of inhibiting postoperative infectious complications. Not only surgery, but interventional examinations such as transrectal prostate biopsy require antibiotics. There are many reports on the efficacy of PAA to inhibit SSI when compared with no use of antibiotics ^11, 12^.

2) Duration of PAA

Considering the two contradictory concepts mentioned above, what we should do next is to determine how to safely decrease antibiotic use without affecting or increasing the SSI occurrence rate. Another thing we need to consider is that long term antibiotic use can cause untreatable infections by antibiotic-resistant strains ^13^. Therefore, this kind of prospective study is a valuable way to learn how to decrease antibiotic use safely.

3) Semi-contaminated

Many guidelines say that surgeries need to be classified according to the extent of pre-surgical pollution, infection, or bacterial colonization. Especially in urological cases, the concept of clean-contaminated operation exists when opening the urinary tract with the possibility of urine dissemination. Preoperative pyuria or bacteriuria should be checked in advance pre-operatively and treated before surgery, including confirmation of absence of such infection in order to decrease the risk of SSI.

4) HoLEP in Japan

Most of the prospective studies on how to decrease antibiotic use, for instance comparisons between 1-day and 3-day prophylactic antibiotic administration (PAA) for urological surgeries such as TUR-P in prospective 2-group or double-blind studies are from western countries ^14^. Moreover, there are the debate that most studies select a 3-day regimen as a comparator. Then Japanese Urological Association (JUA) guideline recommend single-dose or dose within 48 hours for TUEB/HoLEP as hospitalized patients ^15^. Arguably, individual studies need to be designed or undertaken in each country or region with the same or similar medical systems. For instance, one major difference between most western countries versus Korea and Japan is that high volume surgical centers are more common in the west, and thus the number of surgical cases per surgeons is higher in the west than Japan. High volume centers expect shorter surgical times, which can affect SSI occurrence ^7, 16^. These issues support the necessity of our study.

5) Prior studies as evidence for drawing up guidelines

There are several studies regarding the optimal PAA duration period ^17, 18^. However, we need to decrease the variation in patients’ backgrounds in such studies; How many patients with preoperative UTI cases that need to be controlled before surgery. There is no definitive study for comparison between controlled and uncontrolled preoperative UTI caused by retention ^19^ and SSI occurrence after HoLEP. Also, case numbers are limited; so, variations in patient criteria and the surgeons’ experience cannot be ignored.

6) Sample size

This kind of prospective feasibility study needs a setting with the necessary case numbers for analysis. A similar study referred to above had 164 cases for comparison in 2 groups with TUR-P and HoLEP for SSI occurrence, with SSI occurrence after HoLEP being 2/72 cases (2.8%) ^9^. We therefore anticipate 180 cases (90 cases per arm) for a feasibility study with 95% CI for the future design of a prospective double blind non-inferiority study. To our knowledge, no definitive study with one-day PAA for HoLEP has been performed so, we have designed this study as a comparison with 2-days dosing.

7) Study setting

In most retrospective studies, the study period for patients’ enrollment may be different. For instance, comparing TUR-P and HoLEP, HoLEP is a comparatively newer technique leading to the possibility of selection bias for HoLEP surgeons with TUR-P experience and this may influence the results of SSI occurrence. To reduce selection bias, prospective studies where not only patients but also surgeons have similar backgrounds, should be undertaken for definitive conclusions with high evidence level.

8) Significance and problems with prospective studies

Many guidelines refer to prospective studies as providing a high evidence level ^20^. However, prospective randomized studies with intervention may be more difficult to perform ^21^. In Japan, apart from research by medical doctors, publication of scientific papers in the medical field is decreasing. IRB referees need to be strict about study approval and this can make things more complicated for prospective clinical interventional studies. Protocol papers will help researchers plan, design and perform clinical studies. This study describes a comparison study of PAA for HoLEP with 1-day (single dose) and 2-day dosing and help to establish a protocol for PAA taking into account decreasing unnecessary antimicrobial use to prevent the development of AMR.

## Data availability

### Underlying data

All data underlying the results are available as part of the article and no additional source data are required

### Extended data

The consent form and information sheet that will be used in this study is available from Harvard Dataverse

Harvard Dataverse: Extended data. Replication data for Protocol for a comparison study of 1-day versus 2-day prophylactic antibiotic administration in Holmium Laser enucleation of the prostate (HoLEP): a randomized controlled trial https://doi.org/10.7910/DVN/SGTWJN ^8^

License: CC0 1.0 Universal

## References

[1] Çek M Tandoğdu Z Naber K: Antibiotic prophylaxis in urology departments, 2005-2010. *Eur Urol.* 2013;63(2):386–94. 10.1016/j.eururo.2012.09.038 23031676

[2] Wolf JS Jr Bennett CJ Dmochowski RR: Best practice policy statement on urologic surgery antimicrobial prophylaxis. *J Urol.* 2008;179(4):1379–90. 10.1016/j.juro.2008.01.068 18280509

[3] Matsumoto T Kiyota H Matsukawa M: Japanese guidelines for prevention of perioperative infections in urological field. *Int J Urol.* 2007;14(10):890–909. 10.1111/j.1442-2042.2007.01869.x 17880286

[4] Togo Y Shimatani K Hanasaki T: Incidence of genitourinary tract infection following transurethral enucleation with bipolar. *Japanese Journal of Endourology.* 2015;28(2):317–21. 10.11302/jsejje.28.317

[5] Ahyai SA Gilling P Kaplan SA: Meta-analysis of functional outcomes and complications following transurethral procedures for lower urinary tract symptoms resulting from benign prostatic enlargement. *Eur Urol.* 2010;58(3):384–97. 10.1016/j.eururo.2010.06.005 20825758

[6] Togo Y Tanaka S Kanematsu A: Antimicrobial prophylaxis to prevent perioperative infection in urological surgery: a multicenter study. *J Infect Chemother.* 2013;19(6):1093–101. 10.1007/s10156-013-0631-8 23818257

[7] Ishikawa K Maruyama T Kusaka M: [The state of antimicrobial prophylaxis for holmium laser enucleation of the prostate : HoLEP and the results of a questionnaire survey]. *Hinyokika Kiyo.* 2011;57(10):539–43. 22089150

[8] Shigemura K: Replication data for Protocol for a comparison study of 1-day versus 2-day prophylactic antibiotic administration in Holmium Laser enucleation of the prostate (HoLEP): a randomized controlled trial. *Harvard Dataverse, V1.* 2019 10.7910/DVN/SGTWJN PMC6524744 31143442

[9] Jhanwar A Sinha RJ Bansal A: Outcomes of transurethral resection and holmium laser enucleation in more than 60 g of prostate: A prospective randomized study. *Urol Ann.* 2017;9(1):45–50. 10.4103/0974-7796.198904 28216929 PMC5308038

[10] O’Sullivan JW Harvey RT Glasziou PP: Written information for patients (or parents of child patients) to reduce the use of antibiotics for acute upper respiratory tract infections in primary care. *Cochrane Datebase Syst Rev.* 2016;11:CD011360. 10.1002/14651858.CD011360.pub2 27886368 PMC6464519

[11] Morioka H Nagao M Yoshihara S: The first multi-centre point-prevalence survey in four Japanese university hospitals. *J Hosp Infect.* 2018;99(3):325–31. 10.1016/j.jhin.2018.03.005 29549049

[12] Kim DH Bae SR Choi WS: The real practice of antibiotic prophylaxis for prostate biopsy in Korea where the prevalence of quinolone-resistant *Escherichia coli* is high. *Korean J Urol.* 2014;55(9):593–8. 10.4111/kju.2014.55.9.593 25237461 PMC4165922

[13] Levy SB Marshall B: Antibacterial resistance worldwide: causes, challenges and responses. *Nat Med.* 2004;10(12 Suppl):S122–9. 10.1038/nm1145 15577930

[14] Valdevenito Sepúlveda JP: [Antibiotics in transurethral resection of the prostate in patients with low risk of infectious complications: randomized prospective comparative study]. *Arch Esp Urol.* 2004;57(1):48–57. 15112871

[15] Yamamoto S Shigemura K Kiyota H: Essential Japanese guidelines for the prevention of perioperative infections in the urological field: 2015 edition. *Int J Urol.* 2016;23(10):814–824. 10.1111/iju.13161 27531443

[16] Monn MF El Tayeb M Bhojani N: Predictors of Enucleation and Morcellation Time During Holmium Laser Enucleation of the Prostate. *Urology.* 2015;86(2):338–42. 10.1016/j.urology.2015.04.028 26189134

[17] Tsugawa M Hashimoto H Monden K: [Antimicrobial prophylaxis in transurethral resection of the prostate]. *Nihon Hinyokika Gakkai Zasshi.* 1998;89(4):453–9. 10.5980/jpnjurol1989.89.453 9597863

[18] Tsuji Y Okamura K Miyake K: [Antibiotic prophylaxis for transurethral resection of the prostate--comparison of oral administration therapy with intravenous administration therapy]. *Hinyokika Kiyo.* 1993;39(12):1145–52. 7506866

[19] Chen JS Chang CH Yang WH: Acute urinary retention increases the risk of complications after transurethral resection of the prostate: a population-based study. *BJU Int.* 2012;110(11 Pt C):E896–901. 10.1111/j.1464-410X.2012.11471.x 23035623

[20] Roussey-Kesler G Gadjos V Idres N: Antibiotic prophylaxis for the prevention of recurrent urinary tract infection in children with low grade vesicoureteral reflux: results from a prospective randomized study. *J Urol.* 2008;179(2):674–9; discussion 679. 10.1016/j.juro.2007.09.090 18082208

[21] Cardozo L Lisec M Millard R: Randomized, double-blind placebo controlled trial of the once daily antimuscarinic agent solifenacin succinate in patients with overactive bladder. *J Urol.* 2004;172(5 Pt 1):1919–24. 10.1097/01.ju.0000140729.07840.16 15540755

