# Supplementary material for: Protocol for a comparison study of 1-day (single dose) versus 2-day prophylactic antibiotic administration in Holmium Laser enucleation of the prostate (HoLEP): a randomized controlled trial
Source: F1000Res. 2019 May 9;8:161. Originally published 2019 Feb 6. [Version 2] doi: 10.12688/f1000research.17660.2 (PMC6524744; doi:10.12688/f1000research.17660.2)
Supplement: Peer review history

# Peer review: Reviewer response for version 2

**DOI:** 10.5256/f1000research.21093.r48237

**Published:** 2019-05-16

**Authors:** Seung-Ju Lee

**Affiliations:** 1 Department of Urology, St Vincent's Hospital, The Catholic University of Korea, Paldal-gu, Suwon, South Korea

I have no further comments.

I have read this submission. I believe that I have an appropriate level of expertise to confirm that it is of an acceptable scientific standard.

# Peer review: Reviewer response for version 1

**DOI:** 10.5256/f1000research.19311.r47977

**Published:** 2019-05-02

**Authors:** Florian Wagenlehner

**Affiliations:** 1 Clinic & Policlinic for Urology, Pediatric Urology & Andrology, Justus-Liebig University, Giessen, Germany

The authors submitted a protocol studying two different prophylactic regimens in patients undergoing Holmium Laser enucleation of the prostate. The study is randomized.

 The topic of the study is worth while studying, as there are relatively little data especially in laser enucleation of the prostate.

 The study design is well described and set up, including the important in- and exclusion criteria. There is one exclusion criterium leucocyturia, which should be reconsidered, as especially patients with large prostates, might have elevated leucocytes in their urine, and those patients would not be included in the study, although holmium laser enucleation is especially done in patients with large prostates.

I have read this submission. I believe that I have an appropriate level of expertise to confirm that it is of an acceptable scientific standard.

# Response

**Published:** 2019-05-02

**Authors:** Katsumi Shigemura

**Affiliations:** Kobe University Hospital, Japan

Thanks, we added the patients with leucocyturia and reflected on the text (See in Eligibility criteria).

We revised as same way in another part (See in Screening test).

# Peer review: Reviewer response for version 1

**DOI:** 10.5256/f1000research.19311.r44091

**Published:** 2019-04-29

**Authors:** Seung-Ju Lee

**Affiliations:** 1 Department of Urology, St Vincent's Hospital, The Catholic University of Korea, Paldal-gu, Suwon, South Korea

This study protocol aims to establish optimal duration of cefazolin antibiotic prophylaxis prior to HoLEP procedure. The rationale and objective of the study protocol is clearly defined and the overall study design looks appropriate. Regarding details of the method, I have a couple of queries.

  

- First, one-day dosing and single-dose dosing should be distinguished. If you look at your protocol, it's single-dose (CEZ 1g, once per i.v. just before HoLEP/TUEB). However, the study title is 1-day vs. 2-day. Cefazolin is a cephalosporin antibiotic and is time-dependent. Therefore, 1-day (two or three doses per a day) and single-dose are different dosages.
- Second, most studies select a 3-day regimen as a comparator. Why did you choose 2-day? The reason for comparing the 2-day regimen in this study should be more clear.

I have read this submission. I believe that I have an appropriate level of expertise to confirm that it is of an acceptable scientific standard, however I have significant reservations, as outlined above.

# Response

**Published:** 2019-04-30

**Authors:** Katsumi Shigemura

**Affiliations:** Kobe University Hospital, Japan

First, one-day dosing and single-dose dosing should be distinguished. If you look at your protocol, it's single-dose (CEZ 1g, once per i.v. just before HoLEP/TUEB). However, the study title is 1-day vs. 2-day. Cefazolin is a cephalosporin antibiotic and is time-dependent. Therefore, 1-day (two or three doses per a day) and single-dose are different dosages.

(Amendment)

Thanks, our 1-day protocol means single-dose protocol.

Second, most studies select a 3-day regimen as a comparator. Why did you choose 2-day? The reason for comparing the 2-day regimen in this study should be more clear.

(Amendment)

Thanks, but JUA guideline recommend single-dose or dose withing 48 hours (ref: Yamamoto S, Shigemura K, Kiyota H, Wada K, Hayami H, Yasuda M, Takahashi S, Ishikawa K, Hamasuna R, Arakawa S, Matsumoto T; Japanese Research Group for UTI.Essential Japanese guidelines for the prevention of perioperative infections in the urological field: 2015 edition. Int J Urol. 2016 Oct;23(10):814-824.).

# Response

**Published:** 2019-05-02

**Authors:** Katsumi Shigemura

**Affiliations:** Kobe University Hospital, Japan

And my previous comment (single dose) were reflected in the text (See in many places including title)

 Next comment (most studies select a 3-day regimen as a comparator. Why did you choose 2-day? The reason for comparing the 2-day regimen in this study should be more clear.): We reflected on the text (See in 4) HoLEP in Japan in Discussion).

# Response

**Published:** 2019-05-03

**Authors:** Katsumi Shigemura

**Affiliations:** Kobe University Hospital, Japan

I have posted new version of manuscript.
